# Association Between Parental Oral Health Literacy and Oral Health-Related Quality of Life in Children with Autism Spectrum Disorder: A Comparative Cross-Sectional Study

**DOI:** 10.3390/jcm15062266

**Published:** 2026-03-17

**Authors:** Elif Nur Beyaz, Fatih Öznurhan

**Affiliations:** Department of Pediatric Dentistry, Faculty of Dentistry, Sivas Cumhuriyet University, 58140 Sivas, Türkiye; fatihozn@hotmail.com

**Keywords:** autism spectrum disorder, oral health literacy, oral health-related quality of life, pediatric dentistry

## Abstract

**Background/Objectives:** Children with autism spectrum disorder (ASD) may have difficulty maintaining effective and routine oral hygiene practices because of sensory sensitivities, behavioral challenges, and barriers to dental care. These difficulties may adversely affect oral health-related quality of life (OHRQoL). Parental oral health literacy may also influence oral health outcomes in this population. This study compared parental oral health literacy and OHRQoL between children with ASD and typically developing children and examined the association between parental oral health literacy and OHRQoL in the ASD group. Higher POQL scores indicate poorer OHRQoL. **Methods:** This cross-sectional study included 72 children with ASD and 70 typically developing children aged 3–15 years. Data were collected using the Sociodemographic and Oral Health Behaviors Questionnaire, the Turkish version of the Oral Health Literacy Assessment Task for Pediatric Dentistry (TOHLAT-P), and the parent-report Pediatric Oral Health-Related Quality of Life measure (POQL). Group differences were analyzed using the Mann–Whitney U test. Associations were evaluated using Spearman correlation and multivariable linear regression. **Results:** Parental oral health literacy scores were significantly lower in the ASD group than in the control group (26.89 ± 7.94 vs. 31.61 ± 10.98; *p* = 0.002). Significant between-group differences were found in the POQL total score and the social functioning subscale (*p* = 0.025 and *p* = 0.003, respectively). In the ASD group, higher parental oral health literacy was associated with lower POQL total scores (Spearman r = −0.239; *p* = 0.043). In multivariable linear regression, parental oral health literacy remained significantly associated with the POQL total score in children with ASD (B = −0.589; *p* = 0.029; R^2^ = 0.117). **Conclusions:** Parental oral health literacy was associated with OHRQoL in children with ASD, although the explained variance was modest. These findings support the potential value of family-centered oral health education in this population.

## 1. Introduction

Autism Spectrum Disorder (ASD) is a lifelong neurodevelopmental condition characterized by impairments in social interaction and communication, along with restricted and repetitive patterns of behavior. According to the Diagnostic and Statistical Manual of Mental Disorders, Fifth Edition (DSM-5), ASD is primarily characterized by persistent deficits in two core domains: social communication and social interaction, and restricted, repetitive patterns of behavior, interests, or activities [1]. These symptoms typically manifest in early childhood and cause clinically significant impairment in social, occupational, or other important areas of current functioning. Diagnosis is established through clinical observation and behavioral assessment in the absence of a specific biological marker, and the rising prevalence reported in recent years has made ASD a major public health concern [2,3].

Sensory sensitivities, communication difficulties, and challenges in behavioral regulation are commonly observed in individuals with ASD, substantially complicating daily living activities, including the maintenance of oral and dental health. Difficulties with toothbrushing and flossing, food pouching, sensory aversion to toothpaste, and resistance to oral care may increase caries risk in these children [4,5]. Consequently, oral conditions such as dental caries, gingival inflammation, and dental trauma have been reported more frequently in children with ASD, and oral health-related quality of life (OHRQoL) may also be adversely affected [2,6].

Dental treatment processes in children with ASD also present distinct challenges. Heightened sensitivity to sensory stimuli, dental anxiety, and communication limitations can reduce the effectiveness of clinical procedures and, in many cases, necessitate treatment under general anesthesia, creating an additional burden for both families and the healthcare system [7]. Nevertheless, these difficulties may be mitigated through individualized behavioral guidance and adapted dental approaches, including sensory-adapted environments and preparatory strategies such as video modeling [8].

Factors limiting access to oral healthcare among individuals with ASD are not confined to child-specific characteristics. Economic constraints, shortages of trained personnel, inadequate clinical infrastructure, and inequalities in access to healthcare services can further hinder this process. In addition, parental oral health literacy (OHL) plays a critical role in shaping children’s oral hygiene habits, frequency of dental visits, and adherence to preventive practices [4,9].

Parents with limited oral health literacy may have greater difficulty effectively guiding their children’s oral care, which may be associated with poorer oral health outcomes and a greater negative impact on OHRQoL [10,11]. Given the sensory sensitivities and behavioral characteristics commonly observed in children with ASD, parental knowledge and awareness may play an especially important role in maintaining oral care routines at home [5]. However, evidence remains limited regarding how parental oral health literacy relates to OHRQoL specifically in children with ASD compared with typically developing peers.

In this context, evaluating parent-related factors that influence oral health and OHRQoL in children with ASD is of substantial importance. By examining OHRQoL alongside parental oral health literacy in children with ASD and comparing findings with a typically developing control group, the present study aims to inform preventive and supportive oral health strategies tailored to this population. We hypothesized that parental oral health literacy would be lower in the ASD group than in the control group, and that higher parental oral health literacy would be associated with lower POQL scores (better OHRQoL) among children with ASD.

## 2. Materials and Methods

### 2.1. Study Design and Participants

This study was a comparative cross-sectional investigation conducted between two groups: children diagnosed with Autism Spectrum Disorder (ASD) and typically developing children.

The ASD group was formed through parents reached via special education schools with the permission of the Sivas Provincial Directorate of National Education. Parents of children diagnosed with ASD according to DSM-5 criteria were invited to participate. According to DSM-5, ASD is characterized by persistent deficits in social communication and social interaction, together with restricted, repetitive patterns of behavior, interests, or activities, with symptoms present in the early developmental period and causing clinically significant functional impairment.

The control group was selected using convenience sampling from parents of children aged 3–15 years who attended the Department of Pediatric Dentistry, Sivas Cumhuriyet University Faculty of Dentistry, and who had no systemic or neurological disease. Because the control group was recruited from a clinical setting, the possibility of selection bias was considered in the interpretation of the findings. In addition, no individual matching was performed between the ASD and control groups.

Written informed consent was obtained from all parents. A total of 142 participants were included in the study: 72 in the ASD group and 70 in the control group. Sample size was calculated using G*Power software version 3.1.9.7 (Heinrich Heine University Düsseldorf, Düsseldorf, Germany) with a two-tailed independent-samples *t* test. Assuming a medium effect size (Cohen’s d = 0.50), a significance level of α = 0.05, and 80% statistical power, the minimum required total sample size was calculated as 128 participants, corresponding to at least 64 participants per group. The assumption of a medium effect size was based on previous studies suggesting moderate differences in oral health-related quality of life outcomes between pediatric clinical groups and controls [12,13]. Therefore, the final sample size of 142 participants exceeded the minimum requirement. Parents/legal guardians of children aged 3–15 years were included. In the ASD group, parents/legal guardians of children aged 3–15 years with an ASD diagnosis based on DSM-5 criteria were enrolled; in the control group, parents/legal guardians of children aged 3–15 years with no systemic or neurological disease were included. Written informed consent was obtained from all parents/legal guardians, and the questionnaire forms were completed by the parents/legal guardians. Exclusion criteria were as follows: age outside the 3–15-year range, incomplete questionnaire forms, inability of the parent/legal guardian to understand or complete the questionnaire reliably, refusal to provide written informed consent, and the presence of systemic or neurological disease in the control group.

The study was conducted with the approval of the Sivas Cumhuriyet University Non-Interventional Clinical Research Ethics Committee (20 February 2025; No. 2025-02/34). All participants were informed prior to inclusion, and written consent was obtained. Permission to use the TOHLAT-P scale was obtained from the researcher who performed the Turkish adaptation. The POQL scale, being publicly available, was used without additional permission in accordance with the usage conditions stated in the relevant sources.

### 2.2. Data Collection Instruments

Data were collected using a sociodemographic and oral health behaviors form, the TOHLAT-P scale, and the POQL scale.

#### 2.2.1. Sociodemographic and Oral Health Behaviors Form

Parents completed a questionnaire developed by the researchers using items derived from similar studies in the literature and tailored to the objectives of the present study to assess sociodemographic characteristics (e.g., parent age, educational level, income, health insurance) and children’s oral health-related behaviors (e.g., toothbrushing frequency, dental attendance, dietary habits). This questionnaire included 37 items for the ASD group and 32 items for the control group and consisted of multiple-choice, yes/no, and short-answer items. All items included in the control group questionnaire were also included in the ASD group questionnaire; the difference in item number was due to additional ASD-specific questions. Because this questionnaire was designed to collect descriptive background information rather than measure a latent construct, no formal psychometric validation was performed.

#### 2.2.2. POQL (Pediatric Oral Health-Related Quality of Life)

Children’s oral health-related quality of life was assessed using the POQL measure. The instrument was designed to cover preschool, school-age, and preadolescent periods and aims to comprehensively capture the impact of oral health problems on children’s daily life, functioning, and emotional well-being [12]. For the preschool period, only a parent-report form is available, whereas for older children, both parent-report and child self-report versions exist [13]. In the present study, only the parent-report version was used, as children in the ASD group were not expected to complete the questionnaire reliably because of potential difficulties in reading, comprehension, and responding to the items. During the development of the POQL, the intentional oversampling of low-income and minority groups has been described as an important methodological decision aimed at increasing the instrument’s applicability across diverse sociodemographic groups [12]. Multi-stage factor analyses demonstrated that the items cluster into four core domains: physical functioning, role functioning, social functioning, and emotional functioning [14].

POQL scoring is based on assigning separate scores for the frequency of each event and the level of bother it causes; these two scores are multiplied to generate an “impact score” ranging from 0 to 9. If an event did not occur, the item score is recorded as 0. Subscale scores are calculated by summing the impact scores of the relevant items, and the total POQL score is obtained by summing the impact scores of all items. Higher scores indicate a greater negative impact of oral health problems on quality of life [13].

The Turkish adaptation and validity–reliability studies of the POQL were conducted by Yazıcıoğlu et al. in 2018 [14]; following cultural adaptation, linguistic equivalence, content and construct validity, and internal consistency coefficients were evaluated. The findings indicated that the Turkish POQL retained a domain structure consistent with the original instrument and exhibited strong psychometric properties [14]. Oral health-related quality of life measures complement clinical indicators by enabling assessment of the social, emotional, and functional implications of perceived oral health status, and thus constitute an important patient-reported outcome for both research and clinical decision-making [14,15].

#### 2.2.3. TOHLAT-P (Turkish Oral Health Literacy Assessment Task for Pediatric Dentistry)

Parental oral health literacy was assessed using TOHLAT-P, the Turkish adaptation of the HK-OHLAT-P scale originally developed for use in pediatric dentistry [16,17,18]. The Turkish adaptation was performed by Buldur and Oğuz, and its psychometric properties were evaluated through validity and reliability analyses [16].

TOHLAT-P consists of 52 items and evaluates parental oral health literacy across three domains: recognition and naming, literacy and numeracy, and comprehension. The total score ranges from 0 to 52, with higher scores indicating higher parental oral health literacy [16]. Recent studies have reported that TOHLAT-P can be used as a reliable tool to assess oral health literacy among Turkish parents and to examine its associations with clinical indicators [19].

### 2.3. Outcomes

The primary outcome of this study was the association between parental oral health literacy and oral health-related quality of life in children with ASD. Secondary outcomes included comparisons of parental oral health literacy and POQL scores between children with ASD and typically developing controls, as well as the evaluation of associations between sociodemographic characteristics and TOHLAT-P/POQL scores.

### 2.4. Statistical Analysis

Internal consistency of the scales used in the study was assessed using Cronbach’s alpha. Descriptive statistics were then reported. Frequencies and percentages were used for categorical variables, whereas mean, standard deviation, median, minimum, and maximum values were provided for quantitative variables.

Normality was evaluated based on the results of the Kolmogorov–Smirnov and Shapiro–Wilk tests, as well as inspection of histograms, boxplots, and skewness and kurtosis coefficients. Because parametric assumptions were not met, non-parametric tests were used: the Mann–Whitney U test for comparisons between two groups and the Kruskal–Wallis test for comparisons among more than two groups. For multiple comparisons, the Mann–Whitney U test with Bonferroni correction was applied. These tests were used to determine whether variables differed across grouping factors.

Spearman correlation analysis was conducted to examine associations between quantitative variables. The a priori sample size calculation was based on the planned between-group comparison, whereas the regression analyses were performed as adjusted analyses to further examine the association between parental oral health literacy and oral health-related quality of life while controlling for potential confounding factors. Multivariable linear regression analyses were performed separately for the ASD and control groups. In these models, total POQL score was entered as the dependent variable, whereas child age, sex, parental education level, household income, and total TOHLAT-P score were entered as independent variables. Regression coefficients, 95% confidence intervals, *p* values, R^2^, and adjusted R^2^ values were reported. Regression assumptions were evaluated by examining independence of errors using the Durbin–Watson statistic and multicollinearity using tolerance and variance inflation factor (VIF) values. Because multiple statistical comparisons were performed, the primary outcome was predefined, and the remaining analyses were interpreted as secondary analyses. Statistical significance was set at 0.05. All analyses were performed using IBM SPSS Statistics (IBM Corp., Armonk, NY, North America, USA) version 25.0.

## 3. Results

### 3.1. Sample Characteristics

A total of 142 children and their parents were included in the study, comprising 72 children diagnosed with ASD and 70 typically developing children. The mean age of the children was 9.9 ± 2.8 years in the control group and 10.0 ± 3.8 years in the ASD group, with no significant difference between the groups (*p* = 0.612). As shown in Table 1, the groups differed significantly in sex distribution, parental education, and household income (*p* < 0.05). No significant difference was found between the groups in terms of health insurance status (*p* > 0.05). Detailed sociodemographic characteristics are presented in Table 1.

### 3.2. Parental Oral Health Literacy (TOHLAT-P)

When TOHLAT-P total scores were compared, parents in the control group had significantly higher scores than those in the ASD group (*p* = 0.002). In subscale analyses, the “reading comprehension and numeracy” score was significantly higher in the control group (*p* < 0.001), whereas no significant differences were observed between the groups in the other subscales (Table 2).

### 3.3. Children’s Oral Health-Related Quality of Life (POQL)

Analysis of POQL data showed that the social functioning subscale and the total POQL score were significantly higher in the control group than in the ASD group (*p* = 0.003 and *p* = 0.025, respectively) (Table 2). No significant between-group differences were observed in the physical functioning, role functioning, or emotional functioning subscales (*p* > 0.05). Since higher POQL scores indicate poorer oral health-related quality of life, these findings suggest worse parent-reported oral health-related quality of life in the control group for the affected domains.

In the ASD group, TOHLAT-P scores were significantly and negatively correlated with POQL scores for social functioning, emotional functioning, and total POQL score (*p* < 0.05). In contrast, no significant association was observed between TOHLAT-P and POQL in the control group (*p* = 0.921) (Table 3 and Table 4).

### 3.4. Multivariable Linear Regression Analysis

To further evaluate the primary outcome while accounting for potential confounding factors, multivariable linear regression analyses were performed separately for the ASD and control groups, with total POQL score as the dependent variable and child age, sex, parental educational level, household income, and total TOHLAT-P score as independent variables.

In the control group, total TOHLAT-P score was not a significant predictor of total POQL score (B = 0.063, 95% CI: −0.288 to 0.413, *p* = 0.722). Similarly, child age, sex, parental educational level, and household income were not significantly associated with total POQL score (*p* > 0.05). The model showed limited explanatory power (R^2^ = 0.079; adjusted R^2^ = −0.059).

In the ASD group, total TOHLAT-P score was a significant negative predictor of total POQL score (B = −0.589, 95% CI: −1.117 to −0.061, *p* = 0.029). Child age, sex, parental educational level, and household income were not significantly associated with total POQL score (*p* > 0.05). The model explained 11.7% of the variance in total POQL score (R^2^ = 0.117; adjusted R^2^ = −0.012), indicating modest explanatory capacity (Table 5 and Table 6). For the control and ASD regression models, Durbin–Watson values were 1.902 and 1.516, respectively, indicating no major autocorrelation of residuals. Tolerance and VIF values were 1.000 in both models, suggesting no evidence of multicollinearity.

### 3.5. Oral Health Behaviors

As shown in Table 7, toothbrushing responsibility was significantly more often assumed by a parent or caregiver in the ASD group, whereas children in the control group more frequently performed toothbrushing independently (*p* < 0.001). A significant between-group difference was also observed in toothbrushing frequency: non-brushing or infrequent brushing was more common in the ASD group, while brushing at least once daily was more prevalent in the control group (*p* < 0.001). In addition, children with ASD were less likely to have their own toothbrush than controls (*p* = 0.032) and had poorer knowledge regarding the fluoride content of the toothpaste used (*p* < 0.001).

Regarding oral health-related findings reported by parents, gingival bleeding during toothbrushing (*p* = 0.001) and a history of dental trauma (*p* = 0.049) were significantly more frequent in the ASD group.

### 3.6. Use of Dental Services and Clinical Cooperation

When dental service utilization was examined, the proportion of children who had previously visited a dentist was lower in the ASD group than in the control group (*p* < 0.001). Among children with prior dental experience, rates of allowing oral examination and dental treatment were significantly lower in the ASD group (*p* < 0.001). While treatment was largely completed under standard dental unit conditions in the control group, the need for general anesthesia was markedly higher in the ASD group, and treatment could not be performed in some cases (*p* < 0.001). Consistently, the proportion of children able to access and receive the dental care they needed was significantly lower in the ASD group than in the control group (*p* < 0.001).

### 3.7. Perceptions of General Anesthesia

Parental attitudes toward general anesthesia did not differ significantly between groups with respect to believing that general anesthesia has adverse effects (*p* = 0.876) or that it is dangerous and should not be used (*p* = 0.320).

## 4. Discussion

This study compared children with ASD and their typically developing peers in terms of parental oral health literacy (OHL), oral health-related quality of life (OHRQoL), oral health behaviors, and indicators of access to dental services and clinical cooperation while also exploring potential associations between parental literacy and POQL. The primary aim of the study was to examine the association between parental OHL and OHRQoL in children with ASD. ASD is a neurodevelopmental condition that may complicate daily caregiving routines and oral hygiene practices due to sensory sensitivities, difficulties in behavioral regulation, and communication limitations [1]. In this context, children with ASD have been reported to experience oral health problems—such as dental caries, periodontal disease, and dental trauma—more frequently, alongside challenges in cooperating with dental treatment and difficulties in maintaining sustainable home-based oral care [5,8].

The significantly lower parental OHL scores observed in the ASD group are consistent with studies suggesting that parents of children with ASD may have limitations in oral health knowledge, attitudes, and caregiving practices, which in turn may adversely affect the management of their child’s oral health [20,21,22]. Hajiahmadi et al. reported that parents of children with ASD may demonstrate low levels of oral health knowledge and attitudes [20], while Floríndez et al. showed that educational attainment and household income can be important determinants of literacy [22]. Similarly, da Silva et al. emphasized the prominent role of parents in the caregiving process by reporting that children with ASD may be more dependent on family/caregiver support for daily activities such as oral hygiene and that toothbrushing responsibility often rests with the parent [21].

In contrast, several studies have reported comparable parental OHL levels in families of children with ASD or no significant between-group differences [23,24,25]. Moriyama et al., in a sample of typically developing children, found no significant association between parental literacy and clinical oral health indicators and suggested that sociodemographic factors may be more influential [23]. Likewise, Velasco et al. and Bayraktar and Bahadır, working with non-ASD populations, did not demonstrate a direct association between parental literacy and OHRQoL [24,25]. Such discrepancies across studies may stem from methodological and contextual differences, including sample characteristics, socioeconomic composition, access to healthcare, and the sensitivity of the measurement instruments employed.

Parental education and income are critical socioeconomic indicators in the context of diagnosis and management of care needs among children with ASD [26,27]. Lower educational attainment has been reported to hinder parents’ ability to recognize health needs and to access information, potentially reducing engagement with healthcare services [28,29]. In this regard, the positive association between parental education level and OHL reported by Buldur and Oğuz supports the view that higher educational attainment strengthens the ability to process and apply health information [16].

In our study, the significantly lower education and income levels in the ASD group compared with the control group align with reports indicating that these families may have a more disadvantaged sociodemographic profile [28,30]. At the same time, the recruitment of the control group from volunteer parents attending a university hospital may have contributed to relatively higher education levels than would be expected in the general population, introducing potential selection bias. In addition, no individual matching was performed between groups. Therefore, the between-group differences in sex, parental education, and household income should be considered possible sources of confounding, even though multivariable analyses were used to adjust for major sociodemographic factors. Residual confounding cannot be excluded. The literature suggests that the relationship between socioeconomic status and ASD may be heterogeneous, and that healthcare systems and social support structures may influence how this relationship is expressed across settings [31,32].

In our study, the lower POQL total and subscale scores observed in the ASD group may initially appear contradictory given the POQL scoring approach, whereby higher scores reflect greater negative impact. Accordingly, lower scores in the ASD group should not be interpreted as indicating better OHRQoL or better clinical oral health. Because the parent-report POQL focuses on perception-based domains, oral health-specific impacts may be weighted differently by parents in the context of communication difficulties, sensory sensitivities, and a high caregiving burden in ASD, potentially contributing to lower reported scores [21,33]. In addition, children with ASD may have difficulty expressing symptoms such as pain or discomfort, and parental expectations or interpretations of oral-health-related impacts may differ from those in typically developing children. Moreover, pain perception and expression may be atypical in children with ASD and may be more difficult for parents to recognize [34,35]. Thus, given the subjective and multidimensional nature of OHRQoL, POQL scores may not align one-to-one with clinical indicators, and the sensitivity of parent-reported measures may be limited in ASD populations [2,36,37]. Furthermore, the POQL is not a condition-specific instrument developed specifically for children with ASD. Therefore, it may not fully capture oral-health-related impacts that are particularly relevant to this population, and its sensitivity in reflecting the experiences of children with ASD may be limited. Notably, other studies have reported worse OHRQoL in children with ASD, with impacts particularly pronounced in social and emotional domains [33,38]. Differences across studies may be attributable to factors such as reliance on parent-report (without child self-report), sample characteristics, and clinical severity/heterogeneity.

Correlation analyses indicated that the TOHLAT-P total score showed strong, statistically significant correlations with its subscales (r: 0.699–0.845; *p* < 0.001), suggesting that parental OHL reflects a coherent, holistic construct. This finding is consistent with the conceptual model proposed by Buldur and Taşkaya, which defines OHL as a hierarchical competency extending from access to information to its application [24], and with studies reporting similar structural integrity [39,40,41].

The absence of a significant association between OHL and OHRQoL (POQL) in the control group may be related to limited variability due to a low-risk profile and a potential floor effect [39]. In addition, the control group’s recruitment from university hospital volunteers may have resulted in higher awareness levels, thereby attenuating the measurable impact of literacy on quality-of-life outcomes. In contrast, the observed association between OHL and POQL in the ASD group may reflect a greater dependence on parental guidance for oral care in this population. In the multivariable model, higher TOHLAT-P scores were associated with lower total POQL scores in the ASD group, whereas no significant association was found in the control group. This result is consistent with studies suggesting that parental literacy may be associated with children’s quality-of-life outcomes [11,28], while differing from community-based samples such as that of Moriyama et al. in terms of sample characteristics [23].

At the same time, the explanatory power of the multivariable model was modest. In the ASD group, the model explained 11.7% of the variance in total POQL score, indicating limited explanatory capacity. Therefore, although the association between parental OHL and POQL reached statistical significance, it should be interpreted with caution because OHRQoL is a multidimensional and perception-based outcome that cannot be explained by parental OHL alone. Additional factors not included in the present model, such as child behavior, sensory reactivity, dental anxiety, parental stress, previous treatment experiences, and structural barriers to care, are also likely to contribute. Taken together, these findings suggest that efforts to improve oral health outcomes in children with ASD should incorporate not only clinical approaches, but also inclusive strategies aimed at facilitating families’ access to information and reducing caregiving burden and access barriers. In this regard, it may be beneficial to design oral health education materials for parents of children with ASD that are tailored to children’s sensory and communication profiles and are feasible for home implementation, using visually rich, structured, and practice-based formats (e.g., video modeling, social stories, visual routine schedules).

The significantly lower dentist attendance rate in the ASD group (66.7%) compared with the control group (92.9%) (Table 7) may be interpreted as an indicator of barriers to accessing dental services. In this group, lower rates of allowing examination (35.3%) and treatment (15.7%) may be related to the sensory demands of the clinical environment, communication limitations, and difficulties with behavioral cooperation. Similarly, Daneshvar et al. reported that children with ASD exhibited poorer oral health behaviors and more uncooperative behavior during dental examination than healthy peers [42]. As noted by Taşkaya and Buldur [19], factors related to dental anxiety and trust in the dentist may also constitute barriers that hinder cooperation with clinical procedures.

Behavior management challenges in ASD populations may increase the need for sedation/general anesthesia (GA). In our study, completion of 60% of treatments under GA and the inability to perform treatment in 28% of cases due to cooperation difficulties highlight the need for advanced behavior management in this population. The fact that 86.6% of treatments in the control group could be completed under standard dental unit conditions suggests that the observed differences between groups may be linked to diagnosis-related sensory and behavioral components. Finally, the ability to obtain needed treatment remaining at 41.7% in the ASD group indicates that both clinical and system-level barriers may jointly influence the sustainability of appropriate care. This finding may also relate to difficulties in finding experienced specialists and encountering multiple access barriers, as emphasized by Alshihri et al. [43].

This pattern of findings underscores the potential value of integrating sensory-adapted dental practices into routine care to enhance clinical cooperation in children with ASD (e.g., adjustments to light and sound, pre-appointment familiarization with the clinic, graded exposure, and a structured appointment flow). Moreover, early implementation of preventive oral health approaches may contribute to reducing the long-term need for advanced behavior management/GA.

Several methodological limitations should be considered when interpreting these findings. First, the cross-sectional design does not allow causal inference or determination of temporal ordering between variables, raising the possibility of reverse causality in the association between parental literacy and quality of life. Second, the single-center, clinic-based sampling may have introduced selection bias; in particular, higher awareness levels in the control group may have restricted variability and contributed to a weaker statistical association between OHL and OHRQoL. Between-group socioeconomic imbalances (education, income, sex) should be considered potential confounders. Additionally, reliance on parent-reported quality-of-life measures may limit accurate reflection of children’s experiences, particularly in ASD, where pain and sensory responses can be atypical. Furthermore, no clinical oral health indices, such as dmft/DMFT, plaque index, or gingival measures, were collected. This represents a major limitation, because the absence of clinical data restricts interpretation of whether the observed POQL findings reflect actual oral health status or differences in parental perception and reporting. The modest explained variance (R^2^) in regression models is expected for multifactorial, perception-based outcomes and suggests that the effect of OHL may be shaped through mediating variables such as dental anxiety, parental attitudes, and ASD-specific sensory/behavioral characteristics. Finally, the heterogeneity of the ASD group may limit generalizability. Future studies employing larger, multicenter, and longitudinal designs and incorporating clinical oral health indicators (e.g., DMFT, plaque index) alongside parent reports would strengthen interpretability. Moreover, approaches that measure specific components of access barriers (e.g., cost, service availability, appointment processes) in greater detail could more clearly delineate system-level targets for intervention.

## 5. Conclusions

This study suggests that oral health-related quality of life (OHRQoL) in children with ASD is influenced by multiple interacting factors, including parental oral health literacy, diagnosis-related behavioral barriers, and socioeconomic conditions. The lower POQL scores observed in the ASD group should be interpreted cautiously, as they may reflect limitations of parent-reported outcomes and atypical symptom expression rather than better clinical oral health. In the ASD group, higher parental oral health literacy was associated with lower total POQL scores, whereas no significant association was observed in the control group. However, the explanatory power of this association was modest, and the findings should therefore be interpreted with caution. Overall, these results suggest the potential value of approaches that support parental capacity and facilitate access to appropriate oral healthcare for children with ASD. These findings may help inform future family-centered and sensory-adapted oral health strategies, but they should be confirmed in larger, multicenter, and longitudinal studies that also include clinical oral health measures.

## Figures and Tables

**Table 1:** Sociodemographic characteristics.

		Group				*p*-Value
		Control		ASD		
		*n*	%	*n*	%	
Sex	Female	41	58.6%	21	29.2%	0.000 *
	Male	29	41.4%	51	70.8%	
Parental educational level	Primary school	6	8.6%	26	36.1%	0.000 *
	Middle school	15	21.4%	10	13.9%	
	High school	17	24.3%	22	30.6%	
	University	29	41.4%	11	15.3%	
	Master’s/PhD	3	4.3%	3	4.2%	
Monthly Household Income	0–22,000	12	17.1%	29	40.3%	0.002 *
	22,000–45,000	28	40.0%	28	38.9%	
	45,000 and above	30	42.9%	15	20.8%	
Health insurance status	None	4	5.7%	5	6.9%	0.844
	Green Card	4	5.7%	2	2.8%	
	SSI	61	87.1%	64	88.9%	
	Private insurance	1	1.4%	1	1.4%	
Child’s age		**Mean ± SD**		**Mean ± SD**		
		9.9 ± 2.8		10.0 ± 3.8		0.612

Pearson’s chi-square test, Fisher’s exact test, or Mann–Whitney U test, as appropriate. * *p* < 0.05 was considered statistically significant.

**Table 2:** Between-Group Comparison of TOHLAT-P and POQL Scores.

	Control Group		ASD Group		*p*-Value
	Med (Min–Max)	Mean ± SD	Med (Min–Max)	Mean ± SD	
Total TOHLAT-P	35 (9–49)	31.61 ± 10.98	27 (11–43)	26.89 ± 7.94	0.002 *
Recognition and Labeling	9 (0–12)	7.79 ± 3.87	8 (2–12)	7.94 ± 2.64	0.476
Reading Comprehension and Numeracy	17 (4–23)	15.83 ± 5.67	11 (3–22)	11.61 ± 4.42	0.000 *
Cloze and Sentence Ordering	8 (0–14)	8.07 ± 3.68	8 (0–13)	7.38 ± 3.22	0.221
Physical Functioning	25 (0–75)	30.6 ± 19.49	29.17 (0–100)	30.61 ± 22.61	0.951
Role Functioning	10.42 (0–75)	15.36 ± 16.35	0 (0–66.67)	12.73 ± 16.97	0.118
Social Functioning	0 (0–100)	11.27 ± 18.47	0 (0–77.78)	5.32 ± 13.57	0.003 *
Emotional Functioning	16.67 (0–83.33)	20.24 ± 18.54	11.11 (0–83.33)	17.21 ± 20.39	0.130
Total POQL	15 (0–55.83)	18.64 ± 12.38	11.25 (0–66.67)	15.43 ± 14.95	0.025 *

Mann–Whitney U test; * *p* < 0.05.

**Table 3:** Correlations between parental oral health literacy (TOHLAT-P total and subscales) and oral health-related quality of life scores (POQL total and subscales) in the ASD group.

		Total TOHLAT-P	Recognition and Labeling	Reading Comprehension and Numeracy	Cloze and Sentence Ordering	Physical Functioning	Role Functioning	Social Functioning	Emotional Functioning
Recognition and Labeling	*r*	0.721	--						
	*p*	0.000							
Reading Comprehension and Numeracy	*r*	0.824	0.380	--					
	*p*	0.000	0.001						
Cloze and Sentence Ordering	*r*	0.699	0.415	0.318	--				
	*p*	0.000	0.000	0.007					
Physical Functioning	*r*	−0.162	−0.005	−0.204	−0.102	--			
	*p*	0.174	0.966	0.087	0.399				
Role Functioning	*r*	−0.191	−0.009	−0.230	0.011	0.595	--		
	*p*	0.108	0.939	0.054	0.927	0.000			
Social Functioning	*r*	−0.388	−0.287	−0.371	−0.157	0.375	0.556	--	
	*p*	0.001	0.014	0.001	0.191	0.001	0.000		
Emotional Functioning	*r*	−0.270	−0.074	−0.269	−0.227	0.559	0.611	0.482	--
	*p*	0.022	0.538	0.023	0.057	0.000	0.000	0.000	
Total POQL	*r*	−0.239	−0.031	−0.270	−0.145	0.861	0.779	0.589	0.854
	*p*	0.043	0.797	0.023	0.226	0.000	0.000	0.000	0.000

Spearman correlation analysis; *p* < 0.05.

**Table 4:** Correlations between parental oral health literacy (TOHLAT-P total and subscales) and oral health-related quality of life scores (POQL total and subscales) in the control group.

		Total TOHLAT-P	Recognition and Labeling	Reading Comprehension and Numeracy	Cloze and Sentence Ordering	Physical Functioning	Role Functioning	Social Functioning	Emotional Functioning
Recognition and Labeling	r	0.817	--						
	*p*	0.000							
Reading Comprehension and Numeracy	r	0.845	0.612	--					
	*p*	0.000	0.000						
Cloze and Sentence Ordering	r	0.770	0.513	0.443	--				
	*p*	0.000	0.000	0.000					
Physical Functioning	r	−0.041	0.058	0.063	−0.189	--			
	*p*	0.739	0.633	0.603	0.118				
Role Functioning	r	−0.196	−0.146	−0.208	−0.194	0.397	--		
	*p*	0.104	0.229	0.084	0.108	0.001			
Social Functioning	r	0.026	0.101	0.045	−0.053	0.217	0.211	--	
	*p*	0.829	0.403	0.712	0.664	0.071	0.079		
Emotional Functioning	r	−0.030	0.042	−0.102	0.046	0.353	0.265	0.444	--
	*p*	0.802	0.727	0.400	0.705	0.003	0.027	0.000	
Total POQL	r	−0.012	0.080	−0.036	−0.038	0.638	0.527	0.661	0.820
	*p*	0.921	0.510	0.766	0.754	0.000	0.000	0.000	0.000

Spearman correlation analysis; *p* < 0.05.

**Table 5:** Multivariable linear regression analysis of total POQL score in the control group.

Dependent Variable: Total POQL								
Parameter		B	Std. Error	t	*p* Value	95% Confidence Interval		Partial Eta Squared
						Lower Bound	Upper Bound	
Intercept		1.558	10.965	0.142	0.887	−20.375	23.491	0.000
Sex	Female	−0.236	3.400	−0.069	0.945	−7.037	6.565	0.000
	Male	0 ^b^						
Parental educational level	Primary school	9.518	10.657	0.893	0.375	−11.798	30.834	0.013
	Middle school	10.753	8.997	1.195	0.237	−7.243	28.749	0.023
	High school	8.549	8.631	0.991	0.326	−8.715	25.814	0.016
	University	11.095	7.842	1.415	0.162	−4.591	26.781	0.032
	Master’s/PhD	0 ^b^						
Monthly Household Income	0–22,000	3.390	5.008	0.677	0.501	−6.628	13.409	0.008
	22,000–45,000	5.207	4.228	1.232	0.223	−3.250	13.664	0.025
	45,000 and above	0 ^b^						
Child’s age		0.282	0.570	0.495	0.623	−0.858	1.422	0.004
Total TOHLAT-P		0.063	0.175	0.358	0.722	−0.288	0.413	0.002

ᵇ This parameter is set to zero because it is redundant. R Squared = 0.079 (Adjusted R Squared = −0.059).

**Table 6:** Multivariable linear regression analysis of total POQL score in the ASD group.

Dependent Variable: Total POQL								
Parameter		B	Std. Error	t	*p* Value	95% Confidence Interval		Partial Eta Squared
						Lower Bound	Upper Bound	
Intercept		37.157	14.868	2.499	0.015	7.436	66.878	0.092
Sex	Female	4.449	4.203	1.059	0.294	−3.953	12.851	0.018
	Male	0 ^b^						
Parental educational level	Primary school	−3.323	10.307	−0.322	0.748	−23.927	17.281	0.002
	Middle school	0.149	10.673	0.014	0.989	−21.186	21.485	0.000
	High school	1.372	9.814	0.140	0.889	−18.247	20.991	0.000
	University	−2.597	10.053	−0.258	0.797	−22.693	17.499	0.001
	Master’s/PhD	0 ^b^						
Monthly Household Income	0–22,000	−0.768	6.203	−0.124	0.902	−13.167	11.632	0.000
	22,000–45,000	−2.685	5.741	−0.468	0.642	−14.161	8.792	0.004
	45,000 and above	0 ^b^						
Child’s age		−0.466	0.510	−0.915	0.364	−1.485	0.552	0.013
Total TOHLAT-P		−0.589	0.264	−2.230	0.029 *	−1.117	−0.061	0.074

^b^. This parameter is set to zero because it is redundant. R Squared = 0.117 (Adjusted R Squared = −0.012), * *p* < 0.05 was considered statistically significant.

**Table 7:** Oral health-related behaviors in the ASD and control groups.

Oral Health-Related Behaviors		Control Group		ASD Group		*p* Value
		n	%	n	%	
Who is responsible for toothbrushing?	Child (self)	39	55.7%	11	15.3%	0.000 *
	Parent/caregiver	9	12.9%	43	59.7%	
	Both	22	31.4%	13	18.1%	
	None	0	0.0%	5	6.9%	
Toothbrushing frequency	Never	1	1.4%	12	16.7%	0.000 *
	A few times per month	2	2.9%	8	11.1%	
	A few times per week	24	34.3%	33	45.8%	
	Once a day	30	42.9%	18	25.0%	
	More than once a day	13	18.6%	1	1.4%	
Having a personal toothbrush	Yes	68	97.1%	63	87.5%	0.032 *
	No	2	2.9%	9	12.5%	
Fluoride content of the toothpaste	Yes	32	45.7%	14	19.4%	0.000 *
	No	19	27.1%	10	13.9%	
	I don’t know	19	27.1%	40	55.6%	
	Not using toothpaste	0	0.0%	8	11.1%	
Gingival bleeding during toothbrushing	Yes	10	14.3%	28	38.9%	0.001 *
	No	60	85.7%	44	61.1%	
History of dental trauma	Yes	10	14.3%	20	27.8%	0.049 *
	No	60	85.7%	52	72.2%	
Has your child ever visited a dentist?	Yes	65	92.9%	48	66.7%	0.000 *
	No	5	7.1%	24	33.3%	
If yes, did the child allow oral examination?	Yes	62	91.2%	18	35.3%	0.000 *
	No	6	8.8%	33	64.7%	
If yes, did the child allow dental treatment?	Yes	60	89.6%	8	15.7%	0.000 *
	No	7	10.4%	43	84.3%	
If treatment was allowed, how was it delivered?	In the dental unit	58	86.6%	6	12.0%	0.000 *
	Under general anesthesia	9	13.4%	30	60.0%	
	Not performed	0	0.0%	14	28.0%	
Are you able to obtain the dental treatment your child needs?	Yes	66	94.3%	30	41.7%	0.000 *
	No	4	5.7%	42	58.3%	
Do you think general anesthesia has adverse effects on your child?	Yes	33	47.1%	33	45.8%	0.876
	No	37	52.9%	39	54.2%	
Do you think general anesthesia is dangerous and should not be used?	Yes	52	74.3%	48	66.7%	0.320
	No	18	25.7%	24	33.3%	

Pearson’s chi-square test or Fisher’s exact test, as appropriate; * *p* < 0.05.

## Data Availability

The data presented in this study are available on request from the corresponding author.

## Abbreviations

ASD	Autism Spectrum Disorder
OHRQoL	Oral Health-Related Quality of Life
OHL	Oral Health Literacy
TOHLAT-P	Turkish version of the Oral Health Literacy Assessment Task for Pediatric Dentistry
POQL	Pediatric Oral Health-Related Quality of Life
DSM-5	Diagnostic and Statistical Manual of Mental Disorders, 5th Edition
GA	General Anesthesia
SSI	Social Security Institution
HK-OHLAT-P	Hong Kong Oral Health Literacy Assessment Task for Pediatric Dentistry
